# Relationship Between Problematic Social Media Usage and Employee Depression: A Moderated Mediation Model of Mindfulness and Fear of COVID-19

**DOI:** 10.3389/fpsyg.2020.557987

**Published:** 2020-12-16

**Authors:** Mehwish Majeed, Muhammad Irshad, Tasneem Fatima, Jabran Khan, Muhammad Mubbashar Hassan

**Affiliations:** ^1^Faculty of Management Sciences, International Islamic University, Islamabad, Pakistan; ^2^Lahore Business School, University of Lahore, Lahore, Pakistan; ^3^School of Housing Building and Planning University Sains Malaysia, Nilai, Malaysia; ^4^Department of Management and Social Sciences Capital University of Science and Technology, Islamabad, Pakistan

**Keywords:** depression, COVID-19, pandemic (COVID-19), social media, fear, mindfulness

## Abstract

Social media plays a significant role in modern life, but excessive use of it during the COVID-19 pandemic has become a source of concern. Supported by the conservation of resources theory, the current study extends the literature on problematic social media usage during COVID-19 by investigating its association with emotional and mental health outcomes. In a moderated mediation model, this study proposes that problematic social media use by workers during COVID-19 is linked to fear of COVID-19, which is further associated with depression. The current study tested trait mindfulness as an important personal resource that may be associated with reduced fear of COVID-19 despite problematic social media use. The study collected temporally separate data to avoid common method bias. Pakistani employees (*N* = 267) working in different organizations completed a series of survey questionnaires. The results supported the moderated mediation model, showing that problematic social media use during the current pandemic is linked to fear of COVID-19 and depression among employees. Furthermore, trait mindfulness was found to be an important buffer, reducing the negative indirect association between problematic social media use and depression through fear of COVID-19. These results offer implications for practitioners. The limitations of this study and future research directions are also discussed.

## Introduction

The past decade has seen a rapid increase in social media usage, which has stirred debate on its potential benefits and drawbacks (Panahi et al., 2016; Weinstein, 2018). According to some research, social media platforms offer multiple benefits: they satisfy the basic human need for belonging, increase life satisfaction, and reduce loneliness (Zhan et al., 2016; McLaughlin and Sillence, 2018). On the other hand, excessive use of social media has been linked to serious mental health issues such as depression and anxiety (Primack et al., 2017; Reer et al., 2019; Van der Velden et al., 2019). This debate on social media as a double-edged sword, is ongoing (Panahi et al., 2016). However, the recent COVID-19 pandemic has highlighted the negative side of social media by indicating that excessive use is spreading panic, fear, and misinformation regarding COVID-19 among mass populations (Pennycook et al., 2020). In this study, problematic social media usage is defined as; an excessive use of social media regularly, to the extent that it seems difficult to stay away from it (Andreassen et al., 2012). A spike in social media use has been observed during the COVID-19 pandemic as more people rely on it to get the latest COVID-19 related updates (Pennycook et al., 2020). This increase in social media use has enhanced the spread of the so-called COVID-19 “infodemic” (Pulido et al., 2020). The COVID-19 infodemic is defined as an excess of information on COVID-19, some accurate and some fake which makes it difficult for people to find credible sources for guidance and updates (Pulido et al., 2020).

Social media infodemic has always been an issue but this has become an even greater challenge during the COVID-19 pandemic, which has made many people fearful (Pulido et al., 2020). Islam et al. (2020) reported that sharing fake and harmful content on social media platforms is associated with poor mental health. Similarly, another study showed that the social media infodemic was related to panic episodes among social media users (Islam et al., 2020). Fear of COVID-19 can be defined as an unpleasant emotion in which people tend to feel worried that they might get infected by COVID-19 (Ahorsu et al., 2020). As a result of the spread of fear, misinformation, and mental health issues due to problematic social media use, the World Health Organization has also advised people to spend less time on social media sites (Sohrabi et al., 2020). In summary, COVID-19 has created new challenges for the world, making it more important than ever to conduct cyberpsychology (Gao et al., 2020; Guitton, 2020).

Social media can help in disseminating information, which might be useful in dealing with the pandemic, but it is also linked to anxiety and depression (Islam et al., 2020). Depression is defined as a common mental health issue in which the individual feels fatigued as well as sad and loses interest in everything (Kroenke et al., 2001). A recent study monitored posts shared on social media, reporting that social media is overloaded with terrifying information related to COVID-19, such as details of patients who have either lost their lives due to COVID-19 or are currently fighting the disease (Hua and Shaw, 2020). Some users make the situation worse by sharing misleading information on social media (Pennycook et al., 2020). This bombardment of fear-inducing, deceptive information may depress people by spreading waves of fear (Mertens et al., 2020). Some researchers have also warned that fear of COVID-19 is associated with long-term negative outcomes, which might be an additional issue over and above the disease itself (Ren et al., 2020). Hence, it is essential to investigate the antecedents and consequences of fear of COVID-19 (Mertens et al., 2020).

The limited research available on fear of COVID-19 indicates that it is a strong predictor of mental health issues (Ahorsu et al., 2020). People with a fear of COVID-19 may constantly worry about catching the disease, which affects their mental health (Ren et al., 2020). Multiple studies have highlighted a rapid increase in mental health issues since the pandemic hit; however, the extant literature is silent on the predictors of these issues, which warrants immediate inquiry (Zandifar and Badrfam, 2020). Some studies have suggested that problematic social media use and fear of COVID-19 are important factors linked to depression (Pennycook et al., 2020). However, there is still insufficient empirical evidence to support this claim. Thus, based on gaps in the existing literature and the call for research on the negative outcomes of excessive social media use during COVID-19, the current study proposes that problematic social media use during COVID-19 may be related to fear of COVID-19, which is further linked to depression among employees.

**FIGURE 1:**
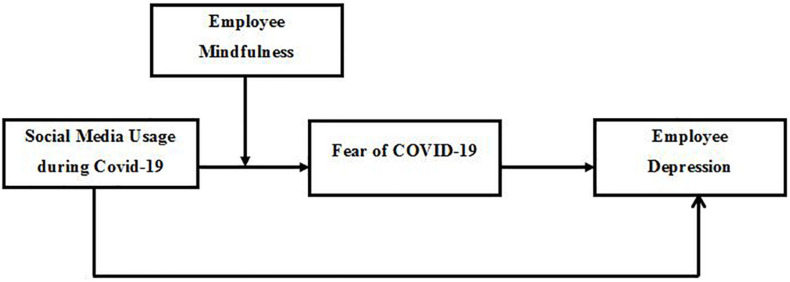
Proposed hypothesized model.

Although a wealth of literature on the adverse psychological outcomes of COVID-19 has been generated within a short time (Lauer et al., 2020), there is a scarcity of research on potential psychological buffers for these outcomes (Duan and Zhu, 2020). It is time to shift focus from problems to solutions, which the world is looking to the research community to deliver (Zhang and Liu, 2020). In this regard, some researchers have recommended meditation practices to reduce mental health issues during the pandemic (Behan, 2020). Similarly, trait mindfulness has also received attention lately due to its extraordinary mental health benefits (Hülsheger et al., 2013). Mindfulness is defined as an extreme form of self-awareness and situation awareness alongside non-judgmental processing of events (Bishop et al., 2004). Existing research has already established the role of mindfulness in reducing the negative effect of stressors (Ireland et al., 2017; Burnett-Zeigler et al., 2018; Sagui-Henson et al., 2018; Montani et al., 2019). However, the role of mindfulness needs to be further explored in the context of the current COVID-19 pandemic (Behan, 2020). Researchers have begun to realize that mindfulness might act as a useful personal resource during a pandemic like COVID-19, which people might use to minimize the fear and negativity associated with COVID-19 (Behan, 2020; Hedderman et al., 2020). Mindfulness refers to a phenomenon in which an individual deliberately engages in non-judgmental processing with respect to present events (Brown and Ryan, 2003; Creswell, 2017). Mindfulness allows people to analyze all the available information in a non-judgmental way and promotes a high sense of self-awareness, which might help them in coping with depression and anxiety (Behan, 2020). People high in trait mindfulness might experience less fear related to COVID-19 than others despite using social media (Hedderman et al., 2020). Hence, the current study proposes that mindfulness weakens the link between problematic social media use and fear of COVID-19, and ultimately with depression. Figure 1 contains the proposed theoretical framework.

## Theory and Hypothesis Development

### Supporting Theory

The current study relies on the conservation of resources (COR) theory (Hobfoll, 1989; Hobfoll et al., 2018) to support the proposed model. This theory discusses the accumulation and preservation of resources. Specifically, people make an effort to accumulate and preserve valuable physical, psychological, financial, and social resources. The threatened or actual loss of these valuable resources causes stress, which gives rise to negative outcomes. In contrast, having other resources available might help in stopping resource loss as a result of exposure to stressors.

Building upon the conservation of resources theory (Hobfoll, 1989), we believe that excessive social media usage during the current pandemic might act as a stressor and thus be linked to adverse outcomes among employees. Excessive exposure to negative information related to COVID-19 on social media threatens employees’ physical resources such as health and life due to the risk of getting the infection, psychological resources such as psychological health by increasing depression and anxiety, social resources such as interpersonal relationships due to social distancing, and financial resources due to the risk of losing one’s job during COVID-19 (Shacham et al., 2020). This threat of resource loss may be associated with stress among employees by developing a fear of catching the infection (Hobfoll et al., 2018; Behan, 2020). According to COR theory, resource loss is more salient than resource gain, and it increases in magnitude and gains momentum over time. Fear induced by stressors might further deplete employees’ resources, resulting in depression (Hobfoll et al., 2018; Majeed and Fatima, 2020; Shacham et al., 2020; van der Velden et al., 2020). Depression represents the most advanced stage of the resource loss cycle, which develops gradually over time and exhausts one’s energy resources (Hobfoll et al., 2018). However, personal resources such as mindfulness may help employees to overcome fear by enhancing self-awarene8ss and helping them in interpreting situations in a non-judgmental way, which can minimize resource loss resulting from the stressor of problematic social media use during COVID-19. Drawing on the COR theory (Hobfoll, 1989; Hobfoll et al., 2018), mindfulness might be considered a personal resource that enables individuals to manage external stressors more effectively (Montani et al., 2019). Furthermore, mindfulness may also help people in gaining further psychological and emotional resources.

### Relationship Between Problematic Social Media Use and Employee Depression

Researchers have reported an increase in social media use during the COVID-19 pandemic (Islam et al., 2020). Social media provides excellent ways to disseminate important information related to COVID-19 and keep people connected in this time of social distancing (Sohrabi et al., 2020). However, studies have found that there are negative outcomes from excessive social media use during the pandemic due to the spread of misinformation (Pennycook et al., 2020). For instance, one study found that the COVID-19-related infodemic, which includes rumors, stigma, and conspiracy theories, is negatively linked to public health (Islam et al., 2020). The negative consequences of social media use cannot be overlooked during the COVID-19 pandemic because conspiracy theories and misinformation about the spread of COVID-19 can be found on most social media sites (Islam et al., 2020). Previous research on problematic social media use has also reported associations with negative outcomes such as depression, poor mental well-being, anxiety, and even suicidal ideation (Primack et al., 2017; Jasso-Medrano and Lopez-Rosales, 2018; Van der Velden et al., 2019). The current COVID-19 pandemic seems to have enhanced the adverse emotional and psychological outcomes of problematic social media use (Pennycook et al., 2020).

Researchers have started to realize that psychological issues related to COVID-19-need immediate attention (Ren et al., 2020). The World Health Organization representative has also advised people to limit social media use during COVID-19 to minimize the chances of panic and mental health issues (World Health Organization [WHO], 2020). It has been noted that social media currently contains content about COVID-19 deaths, patient suffering, and even large numbers of coffins, which causes stress among social media users (Gao et al., 2020; World Health Organization [WHO], 2020). Conservation of resources theory also states that stressful situations threaten and deplete psychological resources, causing stress (Hobfoll, 1989; Hobfoll et al., 2018). It is proposed that excessive use of social media during the COVID-19 pandemic threatens and depletes employees’ valuable resources, leading to stress and ultimately depression. Hence, the current study proposes:

H1: Problematic social media use during COVID-19 is positively linked to employee depression.

### Mediating Role of Fear of COVID-19

As the global death toll due to COIVD-19 continues to rise, many people fear catching COVID-19 (Montemurro, 2020). One of the primary reasons behind this increased fear is the excessive use of social media. Social media platforms have become home to horrific and sometimes fallacious information related to COVID-19 (Islam et al., 2020). Social media users are spreading rumors, conspiracy theories, and even erroneous calculations of COVID-19 cases and deaths, which is spreading fear among the masses (Pennycook et al., 2020).

Another problem is the sharing of disturbing videos on social media in which nurses say goodbye to their families before leaving to treat COVID-19 patients, patient suffering, and coffins on trucks, which seems to enhance fear of COVID-19 (Li et al., 2020). Several other studies have also highlighted that fear of catching the infection is increasing with each coming day, which is linked to psychological health issues (Hong et al., 2020). Fear represents a less intense, smaller initial reaction to the stressor, which can become more severe and intense over time, leading to depression (Hobfoll et al., 2018). These negative outcomes occur due to continuous exposure to stressful news on social media, which steadily depletes personal resources. The extant research also suggests that continuous exposure to stressors consumes employee resources, leading to adverse outcomes that become more severe over time (Majeed and Fatima, 2020).

Fear of catching COVID-19 may be related to mental health issues (Mertens et al., 2020). According to recent studies, people who frequently use social media are more likely to develop a fear of COVID-19, which gives rise to depression, anxiety, and other mental health issues (Pennycook et al., 2020). Due to the severity of these issues, there has been a repeated call to study psychological and mental health problems during COVID-19. Many people are reporting symptoms of mental health problems (Zandifar and Badrfam, 2020). According to COR theory, this increase in mental health issues is due to the depletion of psychological resources (Hobfoll, 1989; Hobfoll et al., 2018). Problematic social media use is a stressor that threatens and depletes employee resources by developing a fear of COVID-19 among a workforce. This fear consumes employees and valuable resources and is linked to depression (Hobfoll, 1989; Hobfoll et al., 2018). Hence, the current study proposes:

H2: Fear of COVID-19 mediates the relationship between problematic social media use during COVID-19 and employee depression.

### Moderating Role of Mindfulness

While medical research on COVID-19 largely focuses on treatments and vaccinations, scholars have also started to raise awareness on maintaining psychological health during this challenging time (Qiu et al., 2020; Wang et al., 2020). The extant literature suggests that problematic social media use during the COVID-19 pandemic is associated with fear (Cinelli et al., 2020; Lum and Tambyah, 2020). This fear develops due to the spread of horrifying information related to COVID-19 (Cinelli et al., 2020; Lum and Tambyah, 2020; Pennycook et al., 2020). To combat a rapid increase in mental health problems, researchers have highlighted the importance of meditation and other mental strengthening activities to keep fear of COVID-19 away (Yanyu et al., 2020). Mindfulness is considered an important personal ability that may help people to avoid experiencing negative emotions (Conversano et al., 2020). An abundance of studies have highlighted the benefits of mindfulness (Donald et al., 2016; Huang et al., 2016; Pang and Ruch, 2019). For instance, a meta-analysis found that mindfulness is negatively related to negative emotions and positively associated with mental health among cancer survivors, as it enables them to suspend judgment and accept their current circumstances (Huang et al., 2016; Behan, 2020; Hedderman et al., 2020; Pecore, 2020). Recent studies have also shown that mindfulness interventions help employees to cope with negative emotions during the pandemic (Behan, 2020; Hedderman et al., 2020; Pecore, 2020). However, little is known about the benefits of trait mindfulness in minimizing negative emotions related to COVID-19 (Conversano et al., 2020). Hence, the current study investigates the role of trait mindfulness in reducing fear of COVID-19 due to problematic social media use. From a COR perspective (Hobfoll, 1989; Hobfoll et al., 2018), trait mindfulness acts as a useful resource that might prevent the depletion of resources due to stressors. Thus, it is proposed that highly mindful employees may experience less fear of COVID-19 due to problematic social media use. Hence, the current study proposes:

H3: Mindfulness moderates the relationship between problematic social media use and fear of COVID-19 in such a way that the relationship will be weaker in the case of higher mindfulness and strong in the case of lower mindfulness.

The current study also proposes a moderated mediation model (Preacher et al., 2007) with a conditional indirect effect on employees’ problematic social media use or depression through fear of COVID-19. Highly mindful employees may be less likely to experience fear despite problematic social media use. Thus, they should be less vulnerable to depression than those who are less mindful. In this way, mindfulness acts as a resource and potential buffer, which might help employees to gain new resources and reduce resource depletion in response to external stressors. The current study proposes that highly mindful employees may experience less fear and depression resulting from problematic social media use during COVID-19. Hence, the present study proposes:

H4: Mindfulness moderates the indirect effect of problematic social media use during COVID-19 on employee depression via fear of COVID-19 in such a way that the indirect effect will be weaker in the case of high mindfulness and stronger in the case of lower mindfulness.

## Materials and Methods

### Sample and Procedure

The current study is quantitative. Physical contact with respondents was not possible due to the COVID-19 lockdown restrictions; therefore, all respondents were contacted online. Furthermore, only currently working employees were considered for participation in the study. This study followed the CHEERIES checklist for e-surveys and the STROBE checklist for time-lagged studies. The researchers’ institutional review board approved the study. Data for all variables were self-reported, which enhances the risk of common method bias. To minimize common method bias in these self-report measures, the data were collected in three time lags with a minimum gap of 7 days between each lag, in line with the recommendations of Podsakoff et al. (2012). Multiple studies have collected time-lagged data to minimize common method bias (Irshad et al., 2020; Majeed and Fatima, 2020). The number of COVID-19 cases in Pakistan was increasing throughout the data collection process and the interval between lags was not that long. Hence, we assumed that there would not be a significant variation in social media use across different lags. Furthermore, we did not find any significant statistical differences in demographic characteristics across the three time lags.

Pakistan reported its first COVID-19 case on February 26, 2020 (Shahid, 2020). The country’s COVID-19 cases began steadily increasing on March 11 (Malik, 2020). The data collection process began on March 20, 2020, and ended on April 23, 2020. A total of 1,865 confirmed cases of COVID-19 had been reported by the end of March, of which 26 patients died (Government of Pakistan, 2020). The virus was spreading fast during the data collection process, with the total number of cases reaching 4,000 on April 7, 2020, and 10,000 on April 22, 2020 (Government of Pakistan, 2020). During the data collection process, the country was in lockdown due to the sudden increase in COVID-19 patients (Shehzad, 2020). According to the official government website on COVID-19 patients in Pakistan, roughly 1,700 confirmed cases were reported in Pakistan between March 23, 2020, and April 25, 2020 (Government of Pakistan, 2020). Pakistan’s nationwide lockdown during the data collection process affected all employees equally, with more than 90% of organizations bound to work from home.

We used a non-probability convenience sampling technique for data collection as the total population of employed individuals in Pakistan is unknown. The authors collected the email addresses of employees working in different organizations through personal contacts and organizations’ official websites. Data were collected from 12 organizations, seven of which were universities, whereas the remaining 5 were in the IT field. Informed consent was obtained from the respondents before participation. The informed consent form explicitly mentioned that participation is voluntary and described the purpose of the study. Although no monetary benefit was given to the participants, they were promised that the survey results would be shared with them upon request. The survey was open enrollment; anyone with the survey URL could participate. We used Google Forms to implement the survey. The authors emailed the informed consent form to potential respondents. The informed consent form contained the study’s purpose and participation criterion along with the URL for Time 1. Email recipients were invited to participate in the study if they met the inclusion criterion. The researchers emailed the URL for Time 2 after a gap of 7 days to all employees who had responded at Time 1. The researchers emailed the URL for Time 3 to employees who had responded at Times 1 and 2. Respondents were able to review their responses before submitting the questionnaire. In accordance with the instructions provided in the CHEERIES checklist for conducting e-surveys, only one response was allowed per email address. This restriction was applied to avoid receiving more than one response from the same respondent. The minimum time required to complete the survey at Time 1 was 10 min, whereas the survey at Time 2 required 4 min, and the survey at Time 3 required a minimum of 6 min.

As the study was time-lagged, the scales were presented in sequence. At Time 1, respondents were asked to provide data on demographics, problematic social media use, and mindfulness. Fear of COVID-19 was measured at Time 2, and employee depression was measured at Time 3. During all three time lags, the respondents were asked whether or not they had contracted COVID-19. The survey comprised three parts: (i) an informed consent form explaining the purpose of the research and ensuring the anonymity of responses; (ii) demographic variables like age, gender, education, and work experience; and (iii) study variables as per the hypothesized model. The inclusion criteria were listed in the informed consent form. Respondents were asked to complete the survey only if they (i) were employed and currently working from home, (ii) had experienced no depressive symptoms or mental health issues before COVID-19, and (iii) had an active social media account.

Twenty-five respondents were in the high-risk group as they were aged 50 and above, but none reported being infected with COVID-19. The confidentiality of the data was fully maintained. The data file was saved in a password-protected folder to which only researchers had access. We assigned a unique I.D. to each respondent and used it to match responses during all three time lags to ensure the anonymity of respondents. We ensured that their responses would be kept confidential and would only be used for the purposes of the study. Three hundred and forty-seven respondents completed the first wave of the study. At Time 2, the 347 employees were contacted again to provide data about their fear of COVID-19, and 312 respondents provided data at Time 2. Finally, these 312 respondents were asked to fill in the questionnaire about depression at Time 3, and 267 responded. These 267 responses were included in the final analysis of the hypothesized model. Thus, the final response rate was 66.7%. The final sample size for all three waves of data collection contained no missing values.

G^∗^Power (version 3.1.9.4) designed by Faul et al. (2009) was employed to assess the sample’s adequacy. The G^∗^Power version 3.1.9.4 has a default value of 0.02 for small effect size, 0.15 for medium effect size, and 0.35 for large effect size, in effect size conventions. For calculating sample size, we selected the *F* test from the test family and selected a statistical test named “Linear multiple regression: Fixed model R2 deviation from zero” from the drop-down menu as recommended by Faul et al. (2009). The number of predictors was set to 3. The default parameters were used (i.e., the medium effect size of 0.15, α level = 0.05, high power of 0.95, number of predictors set to 3) as recommended by Faul et al. (2009). The results revealed an *a priori* sample size of 117 respondents, which is lower than this study’s actual sample size. Subsequently, a *post hoc* power analysis was computed with the same parameters to calculate the power of the collected data (*N* = 267). The power value was 0.99, which is greater than the recommended cutoff value of 0.80 (Cohen, 1992). Based on these *a priori* and *post hoc* analyses, our sample size of 267 is appropriate for testing the proposed model.

The current study is time-lagged, which has the drawback of a lower response rate, as this design requires researchers to approach respondents more than once and most respondents fail to respond at all time lags. Several other time-lagged studies have also shown a very low response rate. For instance, a study in which data was collected in two waves showed a response rate of 49%, only as 162 out of 320 respondents filled in the survey at both time lags (For reference, see Fallman et al., 2019). In a similar time-lagged study conducted with nurses, the response rate dropped from 80% at Time 1 to 43% at Time 2 (Laschinger and Finegan, 2008). The relatively low response rate in the present study is also consistent with the response rate in previous time-lagged studies on COVID-19 in Pakistan (Irshad et al., 2020).

Of the final 267 respondents, 177 were male and 90 were female. 70% of respondents were aged 21–40 years old. 92% had bachelor’s degrees or higher. 63.7% had less than three years of work experience, while the remaining 36.3% had more than 3 years of work experience (see Table 1).

**TABLE 1:** Respondent characteristics.

Variable	Frequency	Percentage
**Gender**		
Male	177	66
Female	90	34
*Age*		
21–30 years	108	40.4
31–40 years	80	30
41–50 years	54	20.2
50 and Above	25	9.4
**Education**		
Below Bachelor	20	7.5
Bachelor	82	30.7
Masters and above	165	61.8
**Experience**		
Less than 1 year	70	26.2
1–3 years	100	37.5
3–5 years	43	16.1
5–7 years	34	12.7
7 and above	20	7.5

*N = 267.*

### Instruments

The questionnaires were adapted and distributed in the English language. The vast majority of employees in Pakistan speak English well (Irshad et al., 2020). Earlier studies have also collected data in English and did not face any language-related issues (e.g., Fatima et al., 2020). The items in each scale were presented sequentially.

#### Problematic Social Media Use

Problematic social media use was measured with a 6-item scale adapted from a previously published source (Andreassen et al., 2012). The scale was constructed using the Bergen Facebook Addiction Scale (BFAS), which contained 18 items on six dimensions, namely salience, relapse, conflict, mood modification, tolerance, and withdrawal. Andreassen et al. (2012) condensed this down to a final six items, one for each dimension, based on high corrected item-total correlations. The final scale used in this study was a unidimensional scale. Each question was answered on a 5-point Likert scale ranging from very rarely (1) to very often (5). The wording of the items was modified to capture social media use during COVID-19. For example: “During COVID-19, how often did you feel an urge to use social media more and more?”. The scale’s Cronbach’s alpha was found to be satisfactory, at 0.83. Other researchers have also used this 6-item scale to measure problematic social media use (Shensa et al., 2017; Kircaburun et al., 2018; Worsley et al., 2018).

#### Fear of COVID-19

Fear of COVID-19 was measured using a 7-item scale recently developed by Ahorsu et al. (2020). Ratings were given on a five-point Likert scale ranging from 1 (strongly disagree) to 5 (strongly agree). A sample item is “I am most afraid of COVID-19” and “My heart races or palpitates when I think about getting COVID-19.” The measure’s Cronbach’s alpha was found to be satisfactory, at 0.84.

#### Mindfulness

The 15-item Mindful Attention and Awareness Scale (MAAS) developed by Brown and Ryan (2003) was used to measure employees’ mindfulness. In the current study, mindfulness is taken as a trait and considered a personal resource. Respondents were asked to rate statements based on a five-point Likert scale ranging from 1 (almost always) to 5 (almost never). A sample item is “I find it difficult to stay focused on what’s happening in the present” (reverse-coded). Cronbach’s alpha was found to be satisfactory, at 0.94. Other studies have also used the Mindful Attention and Awareness Scale (MAAS) to measure employees’ mindfulness (e.g., Hülsheger et al., 2013).

#### Depression

Depression was measured using the brief 9-item Patient Health Questionnaire PHQ-9 developed by Kroenke et al. (2001). The statements were adapted to the context of COVID-19 by asking respondents to rate their depressive symptoms during COVID-19. This variable was measured on a 5-point Likert scale ranging from very rarely (1) to very often (5). A sample item is “Feeling down, depressed, or hopeless.” Cronbach’s alpha for the scale was satisfactory, at 0.86.

Another recent study also used the Patient Health Questionnaire PHQ-9 with a 5-point Likert scale format to measure depression (e.g., Hong et al., 2020). Table 2 shows the prevalence and severity of depression among respondents. The cutoff value for a 5-point Likert scale ranging from 1 to 5 is equivalent to the cutoff criteria for a 5-point Likert scale ranging from 0 to 4 based on percentages. The cutoff criteria provide different ranges for minimal (1–7), mild (8–15), moderate (16–23), moderately severe (24–32), and severe depression (33–45).

**TABLE 2:** Prevalence rates of depressive syndromes.

	No. of respondents	Percentage
Minimal 1–7	0	0
Mild 8–15	8	3.00
Moderate 16–23	51	19.10
Moderately Severe 24–32	114	42.69
Severe 33–45	94	35.21

*N = 267, PHQ-9 scores on 5 point Likert scale.*

### Data Analysis

The Statistical Package for Social Sciences (SPSS) Research Version 21, Process macro by Hayes plugin extension, and AMOS Version 21 were used for data analysis. We confirmed that the data fulfilled all regression assumptions, including linearity, normality, homoscedasticity, multicollinearity, and autocorrelation, before testing the proposed hypotheses. The data was found to be linear, and the error terms were homogenous. Likewise, multicollinearity is not an issue that affects our data because all correlations were well below the cutoff value of 0.70. Additionally, the variance inflation factors (VIF) for all variables were well below the cutoff value of 10 (Myers and Myers, 1990), and all tolerance values lay above the threshold value of 0.2 (Menard, 1995). We also conducted a Durbin-Watson test to confirm that there were no issues regarding autocorrelation. The rule of thumb is that a value of less than 3 but greater than 1 indicates no problems concerning independence of error (Field, 2009, p. 221). In our data, the Durbin-Watson test value is 2.24, which is within the acceptable range. The skewness and kurtosis values for all variables ranged between −1 and +1, the cutoff criteria (Blanca et al., 2013). The skewness values were −0.58 for problematic social media use, −0.69 for fear of COVID-19, −0.22 for depression, and −0.35 for mindfulness. The kurtosis values were −0.21 for problematic social media use, 0.01 for fear of COVID-19, −0.64 for depression, and −0.90 for mindfulness.

The analysis for regression assumptions, means, standard deviations, analysis of variance, correlations, reliability coefficients, and demographic frequency distributions were all conducted using SPSS. Confirmatory factor analysis (CFA) was conducted using AMOS. We used the maximum likelihood method (ML) for estimating parameters in the CFA model. Researchers recommend ML for social sciences research involving Likert scales (Bai and Li, 2016).

The factor loadings for each variable were checked to confirm the convergent validity of the study variables. The factor loadings were greater than 0.4 for all items, which shows that the items load strongly on their respective latent variable. The average variance extracted (AVE), maximum shared variance (MSV), and composite reliability (CR) were calculated to test discriminant validity. Additionally, a four-factor CFA was conducted to further confirm the discriminant validity. For this purpose, we examined the values of model fit indices, including model chi-square (χ^2^), degrees of freedom (df), comparative fit index (CFI), incremental fit index (IFI), Tucker Lewis index (TLI), and the root mean square error of approximation (RMSEA).

Model 7 of the Process macro was used to test the moderated mediation model. The number of bootstrapped samples was set to 5,000, and a 95% confidence interval was specified. The current study utilized a bias-corrected method for constructing confidence intervals.

### Confirmatory Factor Analysis

In line with Anderson and Gerbing’s (1988) recommendation, several CFA tests were also performed to analyze whether the responses matched the hypothesized four-factor model. The results of the CFA are provided in Table 3. For this purpose, five different three-factor models were analyzed by loading the items for two variables onto a single factor. Then, two-factor models were analyzed by loading all the items onto two factors. Finally, the one-factor model was tested by loading all the items onto a single factor. Comparing the results of these three, two, and one-factor models to the four-factor model, the four-factor model yielded better fit indexes, χ*2* = 1064, *df* = 623, χ*2/df* = 1.70, *p* < 05, *CFI* = 0.90, *TLI* = 0.90, *IFI* = 0.90, *RMSEA* = 0.05, all of which are in the acceptable range of model fitness criteria (Hair et al., 2014). The standardized root mean square residual (SRMR) value for the four-factor model is 0.05, which is lower than the cutoff value of.08, thus indicating a good fit (Hu and Bentler, 1999). RMSEA for the four-factor model is.05. Its lower 90% confidence interval (CI) is 0.04 and upper 90% confidence interval (CI) is 0.05, whereas *P* = 0.30. The *p*-value for the close-fitting model is insignificant (*p* > 0.05). The four-factor model yielded better model fit indices compared to the one-factor model. Different alternative models were also tested to check whether the respondents were able to distinguish the different variables from one another. The alternative models showed poorer model fit indices than the hypothesized four-factor model, confirming discriminant validity.

**TABLE 3:** Confirmatory factor analysis and alternative models

Model	χ^2^	df	χ^2^/df	CFI	TLI	IFI	SRMR	RMSEA
**Hypothesized four factors Model (PSMU, FOC, DEP and M)**	**1064**	**623**	**1.70**	**0.90**	**0.90**	**0.90**	**0.05**	**0.05**
One factor (Combine all variables into one factor)	2921	629	4.64	0.51	0.48	0.51	0.15	0.12
Two factor (Combine “PSMU and EM” and “FOC and DEP”)	1952	628	3.11	0.71	0.70	0.72	0.11	0.09
Two factor (Combine “SMU and FOC” and “EM and DEP”)	2270	628	3.61	0.65	0.63	0.65	0.13	0.10
Three factor (Combine PSMU and EM)	1622	626	2.59	0.78	0.77	0.79	0.10	0.08
Three factor (Combine FOC and DEP)	1394	626	2.22	0.83	0.82	0.83	0.08	0.07
Three factor (Combine PSMU and FOC)	1421	626	2.71	0.83	0.82	0.83	0.06	0.07
Three factor (Combine PSMU and DEP)	1434	626	2.91	0.82	0.81	0.83	0.07	0.07
Three factor (Combine EM and FOC)	1621	626	2.59	0.78	0.77	0.79	0.11	0.08

*N = 267. PSMU, Problematic Social Media Use during COVID-19; EM, Employee Mindfulness; FOC, Fear of COVID-19; DEP, Employee Depression. Model fit indices for hypothesized model are given in bold.*

## Results

### Correlation Analysis

Table 4 provides the results for descriptive statistics, average variance extracted (AVE), maximum shared variance (MSV), composite reliabilities (CR), and correlations among the variables of this study. Before computing the correlations, an analysis of variance (ANOVA) test was performed to check the variance in depression and fear of COVID-19 due to demographic variables, i.e., gender, age, education, and experience. ANOVA results for all the demographic variables were found to be non-significant; thus, the demographics relating to respondents were excluded from all further analyses, except the correlation analysis. The problematic use of social media by employees during COVID-19 is significantly correlated with fear of COVID-19 (*r* = 0.38, *p* < 0.01) and depression (*r* = 0.41, *p* < 0.01). Fear of COVID-19 also has a significant positive correlation with employee depression (*r* = 0.45, *p* < 0.01). The mindfulness of employees was found to be significantly negatively correlated with problematic social media use during COVID-19 (*r* = −0.22, *p* < 0.01), fear of COVID-19 (*r* = −0.27, *p* < 0.01), and depression (*r* = −0.12, *p* < 0.05). AVE scores for all variables were greater than 0.50 and lower than the CR, hence establishing convergent validity. Moreover, the AVE scores for all study variables were greater than the MSV scores, thus establishing discriminant validity.

**TABLE 4:** Reliabilities, convergent and discriminant validity, descriptive statistics and intercorrelations.

S. N	Variable	*M*	*SD*	AVE	MSV	CR	1	2	3	4	5	6	7
1	PSMU	3.53	0.80	0.54	0.09	0.81							
2	FOC	3.46	0.76	0.51	0.21	0.84	0.38**						
3	DEP	3.23	0.76	0.52	0.20	0.86	0.41**	0.45**					
4	EM	3.22	0.86	0.59	0.16	0.94	−0.22**	−0.27**	−0.12**				
5	Gender	–	–	–	–	–	0.04	0.09	0.01	0.05			
6	Age	–	–	–	–	–	0.01	0.07	0.09	–0.07	–0.02		
7	Experience	–	–	–	–	–	0.00	0.07	0.15*	–0.01	0.12**	0.45**	
8	Education	–	–	–	–	–	0.09	–0.02	0.03	0.01	–0.05	0.11	0.30**

N = 267; *p < 0.05, **p < 0.01. PSMU, Problematic Social Media Use during COVID-19; FOC, Fear of COVID-19; DEP, Employee Depression; EM, Employee Mindfulness; M, mean; SD, standard deviation; AVE, average variance extracted; MSV, maximum shared variance; CR, composite reliability.

### Hypothesis Testing

Table 5 provides the results for the direct, mediation, moderation, and moderated mediation hypotheses. Hayes (2017) Model 7 of the PROCESS macro was employed to test the hypothesized model. In line with Hypothesis 1, employees’ problematic social media use during COVID-19 was significantly associated with depression (β = 0.26, *p* < 0.01); thus, the H1 of the study was accepted. Furthermore, problematic social media use during COVID-19 was significantly associated with fear of COVID-19 (β = 0.37, *p* < 0.01), and fear of COVID-19 was significantly associated with depression (β = 0.34, *p* < 0.01). The results for the indirect effects confirm the significant mediating role of fear of COVID-19 in the relationship between problematic social media use during COVID-19 and depression (*indirect effect* = 0.12, 95*% CI* with *LL* = 0.07 and *UL* = 0.20). The lower and upper limits of the 95% confidence interval both contain non-zero values. Hence, H2 is also accepted.

**TABLE 5:** Conditional process analysis.

	Unstandardizedβ	SE	LLCI	ULCI
**Mediator variable model**				
PSMU → FOC	0.37**	0.06	0.25	0.48
EM → FOC	−0.15**	0.05	–0.25	–0.10
PSMU x EM → FOC	−0.15**	0.06	–0.27	–0.10
**Dependent variable model**				
FOC → DEP	0.34**	0.06	0.22	44
PSMU → FOC	0.26**	0.05	0.15	0.36
**Indirect effect**				
PSMU → FOC → DEP	0.12*	0.03	0.07	0.20

**Conditional indirect effect (s) of PSMU on DEP through FOC at values of E.M. Mean, ±1 SD**				

	**Effect**	**Boot SE**	**Boot LLCI**	**Boot ULCI**

EM Low -1 SD (2.35)	0.17	0.04	0.10	0.25
EM Mean (3.22)	0.12	0.03	0.07	0.20
EM High + 1 SD (4.09)	0.07	0.03	0.02	0.16
**Index of Moderated Mediation**	–0.05	02	–0.11	–0.02

N = 267, Model 7 results, Bootstrap = 5000, 95% confidence interval. *p < 0.05, **p < 0.01. LL, lower limit; UL, upper limit; SE, standard error. Boot, Bootstrap; PSMU, Problematic Social Media Use during COVID-19; EM, Employee Mindfulness; FOC, Fear of COVID-19; DEP, Employee Depression.

**FIGURE 2:**
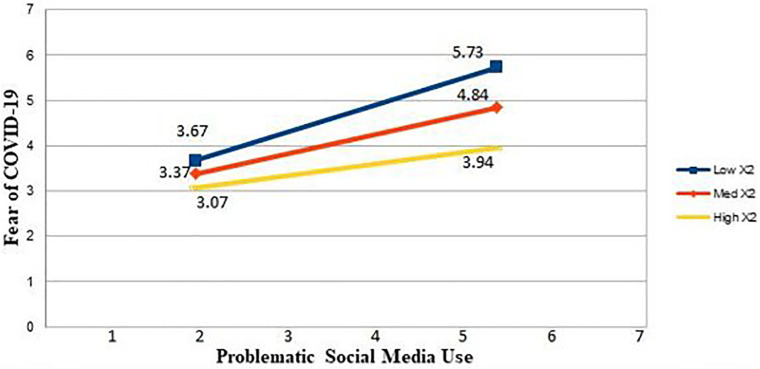
Employee mindfulness as a moderator in the relationship between problematic social media use and fear of COVID-19.

Before testing Hypothesis 3, on problematic social media use during COVID-19 and mindfulness were mean-centered by employing Model 7 of Hayes (2017) Process macro, thus following Aiken et al. (1991) recommendation for testing moderations. The interaction effect of mindfulness and problematic social media use during COVID-19 on fear of COVID-19 was found to be negative and significant (β = −0.15, *p* < 0.01). The moderation graph in Figure 2 shows that mindfulness weakens the relationship between problematic social media use during COVID-19 and fear of COVID-19. Hence, H3 is also supported.

Table 5 also presents results for the conditional indirect effect of employees’ problematic social media use during COVID-19 on depression via fear of COVID-19 at high and low values (±1 *SD* from mean) of mindfulness. The indirect effect of problematic social media use during COVID-19 on depression through fear of COVID-19 weakened at a high level of mindfulness (+1 *SD* from the mean; β = 0.07, *LL 95% CI* = 0.02, *U.L. 95% CI* = 0.16), but grew stronger at a low level of mindfulness (−1 *SD* from the mean; β = 0.17, *LL 95% CI* = 0.10, *UL 95% CI* = 0.25). Additionally, the negative and significant moderated mediation index (*Index* = −0.05, *LL95% CI* = −0.11, *UL95% CI* = −0.02) indicates that mindfulness significantly moderates the indirect effect of problematic social media use during COVID-19 on employee depression via fear of COVID-19. Hence, H4 of the study was also strongly supported by the results.

Table 6 contains a summary of the results for all proposed hypotheses.

**TABLE 6:** Summary of hypothesis results.

H. No	Hypothesis	Support
H1	Problematic social media use during COVID-19 is positively linked to employee depression.	Supported
H2	Fear of COVID-19 mediates the relationship between problematic social media use during COVID-19 and employee depression.	Supported
H3	Employee mindfulness moderates the relationship between problematic social media use and fear of COVID-19 such that the relationship will be weaker in the case of higher employee mindfulness and strong in case of lower employee mindfulness.	Supported
H4	Employee mindfulness moderates the indirect effect of problematic social media use during COVID-19 on employee depression via fear of COVID-19 such that the indirect effect will be weaker in case of high mindfulness and stronger in case of lower mindfulness.	Supported

## Discussion

Despite the potential drawbacks of problematic social media use during COVID-19, there is little research on its association with adverse psychological outcomes, particularly mental health issues, among employees (Pennycook et al., 2020; Zandifar and Badrfam, 2020). The current study aimed to extend research on the negative mental health issues related to COVID-19 by testing a moderated mediation model of problematic social media use and its outcomes. The first objective of the study was to test the association between problematic social media use during COVID-19 and depression among employees. The results supported the first hypothesis by showing that problematic social media use during COVID-19 is strongly associated with an increase in depression among employees (Lum and Tambyah, 2020; Ren et al., 2020). The study’s second aim was to investigate the mediating role of fear of COVID-19 in the relationship between excessive social media use and depression. The results supported our hypothesis that excessive use of social media during COVID-19 is related to fear of COVID-19 among employees, and that fear of COVID-19 is associated with depression. Existing studies also support these results (Li et al., 2020). For instance, several studies found that social media use led to terror and panic during the COVID-19 outbreak (Gao et al., 2020). Prior studies have also found an association between social media use and mental health issues, such as anxiety and depression (Primack et al., 2017; Jasso-Medrano and Lopez-Rosales, 2018; Van der Velden et al., 2019). Similarly, fear of COVID-19 has been linked to severe mental health issues (Ren et al., 2020).

This study further tested the moderating role of mindfulness on the relationship between problematic social media use and fear of COVID-19, and the conditional indirect effect of problematic social media use on employee depression via fear of COVID-19 when mindfulness is high vs. low. The data supported the moderation and moderated mediation hypotheses. This shows that employees with a higher level of mindfulness experience less fear of COVID-19 despite excessive social media use. Hence, they also report experiencing a lower level of depression than employees with a lower level of mindfulness. A few existing studies have come to similar results (Hong et al., 2020). For instance, studies have shown that mindfulness decreases negative emotions experienced during COVID-19 (Conversano et al., 2020). Similarly, there are an abundance of studies on the benefits of mindfulness for mental health (Donald et al., 2016; Huang et al., 2016; Pang and Ruch, 2019).

Despite the potential benefits of social media, problematic use is associated with a chain of negative outcomes that gain momentum over time, ultimately leading to serious outcomes. This study investigated the negative outcomes associated with problematic social media use in the context of a life-threatening pandemic. The study also introduced mindfulness as an important trait to help in dealing with external stressors. This study suggests that employees should refrain from excessive social media use due to its association with negative health outcomes. The study results support the conservation of resources theory in the context of the pandemic by supporting the notion that stressors like excessive and problematic social media use act as a threat to employee resources and are therefore associated with negative outcomes like fear. These negative outcomes then gain momentum and magnitude, and could ultimately take the form of more intense and negative outcomes such as depression. However, personal resources like mindfulness may help employees and protect valuable resources after exposure to such stressors and assist in further resource gains.

### Theoretical Implications

The current study adds to the limited body of knowledge on the psychological outcomes of COVID-19. The use of social media has risen since the onset of the COVID-19 epidemic, and this study showed that problematic social media use during COVID-19 is linked to negative emotional and mental health outcomes. Thus, this study responds to the call for research on the antecedents and consequences of fear of COVID-19. It further contributes to ongoing scholarly discussion that the fear associated with COVID-19 is linked to mental health issues by studying excessive social media use during COVID-19. Additionally, this study identifies trait mindfulness as a useful personal resource by demonstrating that it may help employees to control their fear of COVID-19. Another contribution of this study is that it shines a spotlight on factors linked to depression among employees during the current pandemic. This needs to be addressed quickly, as mental health issues might impede progress and can adversely affect the overall operations of an organization.

### Practical Implications

This study also offers important insights that may have implications for practitioners. Managers could recruit a mental health professional to offer free-of-cost consultations to employees. These consultations may also be provided online, as it is still not safe to meet in person due to the high risk of infection. This could help in resolving employees’ mental health issues. Managers could also increase employees’ awareness about COVID-19, particularly its symptoms and preventive measures so that they are not misled by the misinformation available on social media. One way of doing this is to share research and reports from credible resources on employee email or WhatsApp groups, as accurate information can bust many of the myths linked to COVID-19 and reduce employees’ fear of getting COVID-19. Managers could also conduct regular mindfulness training sessions for their employees, as mindfulness is linked to not only reduced fear of COVID-19 but also lower symptoms of depression and various other mental health issues. Mindfulness training experts could be hired to provide online sessions to employees in their homes.

The findings of the current study could also have implications for policymakers. Policymakers may start campaigns to enhance public awareness of the potential drawbacks of excessive social media use. Government authorities may also wish to create official pages on different social media platforms where people can get accurate information on COVID-19.

### Limitations and Future Research Directions

The current study should be seen in light of its limitations. It investigated the problematic use of social media as a whole rather than the use of any specific platform. Future studies may collect data on the use of specific platforms and their comparative impact on the mental health of users, as each platform has a different user base. This study did not investigate differences in fear of COVID-19 between employees working from home and the office during the lockdown. Multiple organizations required their employees to continue working from the office, including banks and telecom firms. It would be worth studying the difference in fear of catching infection among employees working from home and working from the office in future studies. As another limitation, the scale used to measure problematic social media use did not include the option “Never,” even though some participants might have never “felt an urge to use social media more and more.” Thus, future studies should use a scale containing the “Never” option.

The data of the current study were collected at three different time lags to address common method bias, but this method also has its drawbacks. First, respondents’ social media use, fear of COVID-19, and depression may vary across different time lags. Future studies may wish to collect data for all the variables at all three time lags to compare variations in social media and depression at different time points. Furthermore, this study only highlighted one mental health outcome linked to problematic social media use and fear of COVID-19, namely depression. Future studies might also test for the association between problematic social media use and other psychological health outcomes, such as anxiety, hypertension, and negative emotions, etc. It would also be fruitful to study the link between mental well-being and problematic social media use during COVID-19. This study identified only one dispositional resource, namely mindfulness, which weakens the negative association between problematic social media use during COVID-19 and fear of COVID-19. Future studies might also introduce other personal and situational resources linked to reduced negativity during COVID-19. For instance, it would be useful to test the moderating role of psychological capital, self-efficacy, and family support. Finally, this study only collected data from currently employed individuals, excluding unemployed individuals. Future studies might also seek to collect data from students and unemployed individuals, especially those who lost their job during the pandemic.

Another limitation of this study is the modification of the rating scale for measuring depression. The original scale for depression ranged from 0 and 4; we modified it to have a 1–5 range to maintain a uniform scale for all of the other variables, which were measured on five-point Likert scales ranging from 1 to 5. The Patient Health Questionnaire (PHQ-9) scale for measuring depression severity makes it possible to calculate the prevalence of minimal to severe depression. The cut-off values for minimal to severe depression for the 5-point Likert scale we used might differ slightly from the original measure. This is a potential limitation that could affect the results for depression severity. Future studies might therefore wish to use the original PHQ-9 scale to measure depression.

## Conclusion

COVID-19 is reducing humanity’s economic, physical, social, and now psychological resources. The novel coronavirus continues to infect people worldwide, with the WHO issuing a recent warning that this pandemic is not over and the world will have to face more devastating outcomes. Although social media can help us stay connected with the world in this time of isolation, it is very important to ensure its moderate and controlled use to avoid spreading fear of COVID-19 and prevent further depressive symptoms in people. Building and maintaining psychological and mental health is crucial for preventing adverse outcomes linked to problematic social media use during COVID-19. Mindfulness practices might help in relaxing tension in a difficult environment and may also help in strengthening people’s mental ability to deal with the fear of COVID-19 and other mental health issues.

## Data Availability Statement

The raw data supporting the conclusions of this article will be made available by the authors, without undue reservation.

## Ethics Statement

The studies involving human participants were reviewed and approved by the departmental Ethics Approval Committee at The University of Lahore (UOL). The Faculty of Management Sciences, Lahore Business School UOL Research Ethics Board, reviewed “Detrimental Health Outcomes of Social Media Usage during COVID-19 Outbreak: The Moderating Role of Mindfulness” research proposal and considers the procedures, as described by the applicant, to conform to the University’s ethical standards and university guidelines. Moreover, the participation in the survey was voluntary, and study participants were first explained about the details of the project. It was assured to them that their responses will be kept in strict anonymity and will be reported as aggregate results. Written informed consent for participation was not required for this study in accordance with the national legislation and the institutional requirements.

## Author Contributions

All authors listed have made a substantial, direct and intellectual contribution to the work, and approved it for publication.

## Conflict of Interest

The authors declare that the research was conducted in the absence of any commercial or financial relationships that could be construed as a potential conflict of interest.

## References

[B1] Ahorsu D. K. Lin C. Y. Imani V. Saffari M. Griffiths M. D. Pakpour A. H. (2020). The fear of COVID-19 scale: development and initial validation. *Intern. J. Ment. Health Addict.* 18 1–9. 10.1007/s11469-020-00270-8 32226353 PMC7100496

[B2] Aiken L. S. West S. G. Reno R. R. (1991). *Multiple Regression: Testing and Interpreting Interactions.* Newbury Park, CA: Sage Publications.

[B3] Anderson J. C. Gerbing D. W. (1988). Structural equation modeling in practice: a review and recommended two-step approach. *Psychol. Bull.* 103 411–423. 10.1037/0033-2909.103.3.411

[B4] Andreassen C. S. Torsheim T. Brunborg G. S. Pallesen S. (2012). Development of a Facebook addiction scale. *Psychol. Rep.* 110 501–517. 10.2466/02.09.18.PR0.110.2.501-517 22662404

[B5] Bai J. Li K. (2016). Maximum likelihood estimation and inference for approximate factor models of high dimension. *Rev. Econ. Statist.* 98 298–309. 10.1162/REST_a_00519 8636577

[B6] Behan C. (2020). The benefits of meditation and mindfulness practices during times of crisis such as Covid-19. *Irish J. Psychol. Med.* 37 1–8. 10.1017/ipm.2020.38 PMC7287297 32406348

[B7] Bishop S. R. Lau M. Shapiro S. Carlson L. Anderson N. D. Carmody J. (2004). Mindfulness: a proposed operational definition. *Clin. Psychol. Sci. Pract.* 11 230–241. 10.1093/clipsy.bph077

[B8] Blanca M. J. Arnau J. López-Montiel D. Bono R. Bendayan R. (2013). Skewness and kurtosis in real data samples. *Methodology* 9 78–84. 10.1027/1614-2241/a000057

[B9] Brown K. W. Ryan R. M. (2003). The benefits of being present: mindfulness and its role in psychological well-being. *J. Pers. Soc. Psychol.* 84 822–848. 10.1037/0022-3514.84.4.822 12703651

[B10] Burnett-Zeigler I. E. Waldron E. M. Hong S. Yang A. Wisner K. L. Ciolino J. D. (2018). Accessibility and feasibility of using technology to support mindfulness practice, reduce stress and promote long term mental health. *Complement. Therap. Clin. Pract.* 33 93–99. 10.1016/j.ctcp.2018.09.001 30396633

[B11] Cinelli M. Quattrociocchi W. Galeazzi A. Valensise C. M. Brugnoli E. Schmidt A. L. (2020). The COVID-19 social media infodemic. *arXiv preprint* arXiv:2003.05004. 10.1038/s41598-020-73510-5 PMC7538912 33024152

[B12] Cohen J. (1992). A power primer. *Psychol. Bull.* 112 155–159. 10.1037/0033-2909.112.1.155 19565683

[B13] Conversano C. Di Giuseppe M. Miccoli M. Ciacchini R. Gemignani A. Orrù G. (2020). Mindfulness, age and gender as protective factors against psychological distress during COVID-19 pandemic. *Front. Psychol.* 11:1900. 10.3389/fpsyg.2020.01900 33013503 PMC7516078

[B14] Creswell J. D. (2017). Mindfulness interventions. *Annu. Rev. Psychol.* 68 491–516. 10.1146/annurev-psych-042716-051139 27687118

[B15] Donald J. N. Atkins P. W. Parker P. D. Christie A. M. Ryan R. M. (2016). Daily stress and the benefits of mindfulness: examining the daily and longitudinal relations between present-moment awareness and stress responses. *J. Res. Pers.* 65 30–37. 10.1016/j.jrp.2016.09.002

[B16] Duan L. Zhu G. (2020). Psychological interventions for people affected by the COVID-19 epidemic. *Lancet Psychiatr.* 7 300–302. 10.1016/S2215-0366(20)30073-0 PMC7128328 32085840

[B17] Fallman S. L. Jutengren G. Dellve L. (2019). The impact of restricted decision-making autonomy on health care managers’ health and work performance. *J. Nurs. Manag.* 27 706–714. 10.1111/jonm.12741 30565780

[B18] Fatima T. Majeed M. Jahanzeb S. (2020). Supervisor undermining and submissive behavior: shame resilience theory perspective. *Eur. Manag. J.* 38 191–203. 10.1016/j.emj.2019.07.003

[B19] Faul F. Erdfelder E. Buchner A. Lang A. G. (2009). Statistical power analyses using G^∗^ Power 3.1: tests for correlation and regression analyses. *Behav. Res. Methods* 41 1149–1160. 10.3758/BRM.41.4.1149 19897823

[B20] Field A. P. (2009). *Discovering Statistics Using SPSS: (and Sex, Drugs and Rock “n” Roll)*, 3rd Edn, Los Angeles, CA: Sage.

[B21] Gao J. Zheng P. Jia Y. Chen H. Mao Y. Chen S. (2020). Mental health problems and social media exposure during COVID-19 outbreak. *PLoS One* 15:e0231924. 10.1371/journal.pone.0231924 32298385 PMC7162477

[B22] Government of Pakistan (2020). *Covid-19 Cases in Pakistan.* Available online at: http://covid.gov.pk/stats/pakistan (accessed June 3, 2020).

[B23] Guitton M. J. (2020). Cyberpsychology research and COVID-19. *Comput. Huma. Behav.* 111:106357. 10.1016/j.chb.2020.106357 32362721 PMC7194741

[B24] Hair J. F. Black W. C. Babin B. J. Anderson R. E. (2014). *Multivariate Data Analysis: Pearson New International Edition.* Upper Saddle River, NJ: Pearson Education Limited.

[B25] Hayes A. F. (2017). *Introduction to Mediation, Moderation, and Conditional Process Analysis: A Regression-Based Approach.* New York, NY: Guilford Press.

[B26] Hedderman E. O’Doherty V. O’Connor S. (2020). Mindfulness moments for clinicians in the midst of a pandemic. *Irish J. Psychol. Med.* 37 1–14. 10.1017/ipm.2020.59 32434620 PMC7276502

[B27] Hobfoll S. E. (1989). Conservation of resources: a new attempt at conceptualizing stress. *Am. Psychol.* 44 513–524. 10.1037/0003-066X.44.3.513 2648906

[B28] Hobfoll S. E. Halbesleben J. Neveu J. P. Westman M. (2018). Conservation of resources in the organizational context: the reality of resources and their consequences. *Annu. Rev. Organ. Psychol. Organ. Behav.* 5 103–128. 10.1146/annurev-orgpsych-032117-104640

[B29] Hong S. Ai M. Xu X. Wang W. Chen J. Zhang Q. (2020). Immediate psychological impact on nurses working at 42 government-designated hospital during COVID-19 outbreak in china: a cross-sectional study. *Nurs. Outlook* 10.1016/j.outlook.2020.07.007 [Epub ahead of print], 32919788 PMC7368912

[B30] Hu L. T. Bentler P. M. (1999). Cutoff criteria for fit indexes in covariance structure analysis: conventional criteria versus new alternatives. *Struct. Equ. Model.* 6 1–55. 10.1080/10705519909540118

[B31] Hua J. Shaw R. (2020). Corona virus (COVID-19) “Infodemic” and emerging issues through a data lens: the case of China. *Intern. J. Environ. Res. Public Health* 17:2309 10.3390/ijerph17072309 PMC7177854 32235433

[B32] Huang H. P. He M. Wang H. Y. Zhou M. (2016). A meta-analysis of the benefits of mindfulness-based stress reduction (MBSR) on psychological function among breast cancer (B.C.) survivors. *Breast Cancer* 23 568–576. 10.1007/s12282-015-0604-0 25820148

[B33] Hülsheger U. R. Alberts H. J. Feinholdt A. Lang J. W. (2013). Benefits of mindfulness at work: the role of mindfulness in emotion regulation, emotional exhaustion, and job satisfaction. *J. Appl. Psychol.* 98:310. 10.1037/a0031313 23276118

[B34] Ireland M. J. Clough B. Gill K. Langan F. O’Connor A. Spencer L. (2017). A randomized controlled trial of mindfulness to reduce stress and burnout among intern medical practitioners. *Med. Teach.* 39 409–414. 10.1080/0142159X.2017.1294749 28379084

[B35] Irshad M. Khattak S. A. Hassan M. M. Majeed M. Bashir S. (2020). How perceived threat of Covid-19 causes turnover intention among Pakistani nurses: a moderation and mediation analysis. *Intern. J. Ment. Health Nurs.* 10.1111/inm.12775 [Epub ahead of print], 32779356 PMC7436368

[B36] Islam M. S. Sarkar T. Khan S. H. Mostofa Kamal A. H. Hasan S. M. Kabir A. (2020). COVID-19-related infodemic and its impact on public health: a global social media analysis. *Am. J. Trop. Med. Hyg.* 103 1621–1629. 10.4269/ajtmh.20-0812 32783794 PMC7543839

[B37] Jasso-Medrano J. L. Lopez-Rosales F. (2018). Measuring the relationship between social media use and addictive behavior and depression and suicide ideation among university students. *Comput. Hum. Behav.* 87 183–191. 10.1016/j.chb.2018.05.003

[B38] Kircaburun K. Jonason P. K. Griffiths M. D. (2018). The dark tetrad traits and problematic social media use: the mediating role of cyberbullying and cyberstalking. *Pers. Individ. Differ.* 135 264–269. 10.1016/j.paid.2018.07.034

[B39] Kroenke K. Spitzer R. L. Williams J. B. (2001). The PHQ-9: validity of a brief depression severity measure. *J. Gen. Intern. Med.* 16 606–613. 10.1046/j.1525-1497.2001.016009606.x 11556941 PMC1495268

[B40] Laschinger H. K. S. Finegan J. (2008). Situational and dispositional predictors of nurse manager burnout: a time-lagged analysis. *J. Nurs. Manag.* 16 601–607. 10.1111/j.1365-2834.2008.00904.x 18558930

[B41] Lauer S. A. Grantz K. H. Bi Q. Jones F. K. Zheng Q. Meredith H. R. (2020). The incubation period of coronavirus disease 2019 (COVID-19) from publicly reported confirmed cases: estimation and application. *Ann. Intern. Med.* 172 577–582. 10.7326/M20-0504 32150748 PMC7081172

[B42] Li L. Zhang Q. Wang X. Zhang J. Wang T. Gao T. L. (2020). Characterizing the propagation of situational information in social media during COVID-19 epidemic: a case study on weibo. *IEEE Trans. Comput. Soc. Syst.* 7 556S–562S. 10.1109/TCSS.2020.2980007

[B43] Lum L. H. W. Tambyah P. A. (2020). Outbreak of COVID-19-an urgent need for good science to silence our fears? *Singapore Med. J.* 61 55–57. 10.11622/smedj.2020018 32052064 PMC7052000

[B44] Majeed M. Fatima T. (2020). Impact of exploitative leadership on psychological distress: a study of nurses. *J. Nurs. Manag.* 28 1713–1724. 10.1111/jonm.13127 32772432

[B45] Malik A. (2020). *76 Suspected Coronavirus Patients Reported in Punjab.* Available online at: https://www.thenews.com.pk/print/627575-76-suspected-coronavirus-patients-reported-in-punjab

[B46] McLaughlin C. J. Sillence E. (2018). Buffering against academic loneliness: The benefits of social media-based peer support during postgraduate study. *Active Learn. High. Educ.* 19 1–14. 10.1177/1469787418799185

[B47] Menard S. (1995). *Applied Logistic Regression Analysis. (Sage University Paper Series on Quantitative Applications in the Social Sciences, Series no. 106)*, 2nd Edn, Thousand Oaks, CA: Sage.

[B48] Mertens G. Gerritsen L. Duijndam S. Salemink E. Engelhard I. M. (2020). Fear of the coronavirus (COVID-19): predictors in an online study conducted in March 2020. *J. Anxiety Disord.* 74:102258. 10.1016/j.janxdis.2020.102258 32569905 PMC7286280

[B49] Montani F. Setti I. Sommovigo V. Courcy F. Giorgi G. (2019). Who responds creatively to role conflict? evidence for a curvilinear relationship mediated by cognitive adjustment at work and moderated by mindfulness. *J. Bus. Psychol.* 35 621–641. 10.1007/s10869-019-09644-9

[B50] Montemurro N. (2020). The emotional impact of COVID-19: from medical staff to common people. *Brain Behav. Immun.* 87 23–24. 10.1016/j.bbi.2020.03.032 32240766 PMC7138159

[B51] Myers R. H. Myers R. H. (1990). *Classical and Modern Regression with Applications*, Vol. 2 Belmont, CA: Duxbury Press.

[B52] Panahi S. Watson J. Partridge H. (2016). Social media and physicians: exploring the benefits and challenges. *Health Inform. J.* 22 99–112. 10.1177/1460458214540907 25038200

[B53] Pang D. Ruch W. (2019). Fusing character strengths and mindfulness interventions: benefits for job satisfaction and performance. *J. Occup. Health Psychol.* 24 150–162. 10.1037/ocp0000144 30714812

[B54] Pecore J. L. (2020). Teaching mindfulness for pandemic times. *Curriculum and Teaching Dialogue* 22 163–167.

[B55] Pennycook G. McPhetres J. Zhang Y. Lu J. G. Rand D. G. (2020). Fighting COVID-19 misinformation on social media: experimental evidence for a scalable accuracy-nudge intervention. *Psychol. Sci.* 31 770–780. 10.1177/0956797620939054 32603243 PMC7366427

[B56] Podsakoff P. M. MacKenzie S. B. Podsakoff N. P. (2012). Sources of method bias in social science research and recommendations on how to control it. *Annu. Rev. Psychol.* 63 539–569. 10.1146/annurev-psych-120710-100452 21838546

[B57] Preacher K. J. Rucker D. D. Hayes A. F. (2007). Addressing moderated mediation hypotheses: theory, methods, and prescriptions. *Multivar. Behav. Res.* 42 185–227. 10.1080/00273170701341316 26821081

[B58] Primack B. A. Shensa A. Escobar-Viera C. G. Barrett E. L. Sidani J. E. Colditz J. B. (2017). Use of multiple social media platforms and symptoms of depression and anxiety: a nationally-representative study among U.S. young adults. *Comput. Hum. Behav.* 69 1–9. 10.1016/j.chb.2016.11.013 PMC13268370 42305949

[B59] Pulido C. M. Villarejo-Carballido B. Redondo-Sama G. Gómez A. (2020). COVID-19 infodemic: more retweets for science-based information on coronavirus than for false information. *Intern. Sociol.* 2020:0268580920914755.

[B60] Qiu J. Shen B. Zhao M. Wang Z. Xie B. Xu Y. (2020). A nationwide survey of psychological distress among Chinese people in the COVID-19 epidemic: implications and policy recommendations. *Gen. Psychiatry* 33:e100213. 10.1136/gpsych-2020-100213 32215365 PMC7061893

[B61] Reer F. Tang W. Y. Quandt T. (2019). Psychosocial well-being and social media engagement: the mediating roles of social comparison orientation and fear of missing out. *New Med. Soc.* 21 1486–1505. 10.1177/1461444818823719

[B62] Ren S. Y. Gao R. D. Chen Y. L. (2020). Fear can be more harmful than the severe acute respiratory syndrome coronavirus 2 in controlling the corona virus disease 2019 epidemic. *World J. Clin. Cases* 8 652–657. 10.12998/wjcc.v8.i4.652 32149049 PMC7052559

[B63] Sagui-Henson S. J. Levens S. M. Blevins C. L. (2018). Examining the psychological and emotional mechanisms of mindfulness that reduce stress to enhance healthy behaviours. *Stress Health* 34 379–390. 10.1002/smi.2797 29431918

[B64] Shacham M. Hamama-Raz Y. Kolerman R. Mijiritsky O. Ben-Ezra M. Mijiritsky E. (2020). COVID-19 factors and psychological factors associated with elevated psychological distress among dentists and dental hygienists in Israel. *Intern. J. Environ. Res. Public Health* 17:2900. 10.3390/ijerph17082900 32331401 PMC7215275

[B65] Shahid A. (2020). *Two Coronavirus Cases Confirmed in Pakistan.* Available online at: https://www.pakistantoday.com.pk/2020/02/26/sindh-health-two-coronavirus-cases-confirmed-in-pakistan-confirms-first-coronavirus-case-in-karachi/ (accessed June 12, 2020).

[B66] Shehzad R. (2020). *Countrywide Lockdown Stretched Till May 9.* Available online at: https://tribune.com.pk/story/2206167/countrywide-lockdown-stretched-till-may-9 (accessed June 5, 2020).

[B67] Shensa A. Escobar-Viera C. G. Sidani J. E. Bowman N. D. Marshal M. P. Primack B. A. (2017). Problematic social media use and depressive symptoms among U.S. young adults: a nationally-representative study. *Soc. Sci. Med.* 182 150–157. 10.1016/j.socscimed.2017.03.061 28446367 PMC5476225

[B68] Sohrabi C. Alsafi Z. O’Neill N. Khan M. Kerwan A. Al-Jabir A. (2020). World Health Organization declares global emergency: a review of the 2019 novel coronavirus (COVID-19). *Intern. J. Surg.* 76 71–76. 10.1016/j.ijsu.2020.02.034 32112977 PMC7105032

[B69] Van der Velden P. G. Setti I. van der Meulen E. Das M. (2019). Does social networking sites use predict mental health and sleep problems when prior problems and loneliness are taken into account? A population-based prospective study. *Comput. Hum. Behav.* 93 200–209. 10.1016/j.chb.2018.11.047

[B70] van der Velden P. G. Slachtofferhulp C. C. F. Das M. van Loon P. Bosmans M. (2020). Anxiety and depression symptoms, and lack of emotional support among the general population before and during the COVID-19 pandemic. A prospective national study on prevalence and risk factors. *J. Affect. Disord.* 277 540–548. 10.1016/j.jad.2020.08.026 32889378 PMC7438386

[B71] Wang C. Pan R. Wan X. Tan Y. Xu L. Ho C. S. (2020). Immediate psychological responses and associated factors during the initial stage of the 2019 coronavirus disease (COVID-19) epidemic among the general population in China. *Intern. J. Environ. Res. Public Health* 17:1729. 10.3390/ijerph17051729 32155789 PMC7084952

[B72] Weinstein E. (2018). The social media see-saw: positive and negative influences on adolescents’ affective well-being. *New Media Soc.* 20 3597–3623. 10.1177/1461444818755634

[B73] World Health Organization [WHO] (2020). *Coronavirus Disease 2019 (COVID-19): Situation Report, 72.* Geneva: WHO.

[B74] Worsley J. D. McIntyre J. C. Bentall R. P. Corcoran R. (2018). Childhood maltreatment and problematic social media use: the role of attachment and depression. *Psychiatry Res.* 267 88–93. 29886276 10.1016/j.psychres.2018.05.023

[B75] Yanyu J. Xi Y. Huiqi T. Bangjiang F. Bin L. Yabin G. (2020). Meditation-based interventions might be helpful for coping with the Coronavirus disease 2019 (COVID-19). 10.31219/osf.io/f3xzq

[B76] Zandifar A. Badrfam R. (2020). Iranian mental health during the COVID-19 epidemic. *Asian J. Psychiatry* 51:101990. 10.1016/j.ajp.2020.101990 32163908 PMC7128485

[B77] Zhan L. Sun Y. Wang N. Zhang X. (2016). Understanding the influence of social media on people’s life satisfaction through two competing explanatory mechanisms. *Aslib J. Inform. Manag.* 68 347–361. 10.1108/ajim-12-2015-0195

[B78] Zhang L. Liu Y. (2020). Potential interventions for novel coronavirus in China: a systemic review. *J. Med. Virol.* 92 479–490. 10.1002/jmv.25707 32052466 PMC7166986

